# Cytokine-Like Protein 1 (CYTL1) as a Key Target of M-Stage Immune Infiltration in Stomach Adenocarcinoma

**DOI:** 10.1155/2023/2926218

**Published:** 2023-02-13

**Authors:** Fang Zhou, QianXia Lin, ZiHan Zheng

**Affiliations:** ^1^Vascular Breast Surgery, Jiangxi Provincial People's Hospital, Nanchang, Jiangxi 330006, China; ^2^Jiangxi University of Traditional Chinese Medicine, Nanchang, Jiangxi 330006, China; ^3^Department of Gastrointestinal Surgery, Jiangxi Provincial People's Hospital, Nanchang, Jiangxi 330006, China

## Abstract

**Background:**

Stomach adenocarcinoma (STAD) has an extremely high fatality rate worldwide, and survival after metastasis is extremely poor. Cytokine-like protein 1 (CYTL1) has prognostic significance in various tumors. We aimed to explore the impact and underlying molecular mechanisms of CYTL1 in STAD through bioinformatics analysis.

**Methods:**

We used R software to analyze CYTL1 expression in STAD samples (*n* = 375) and normal samples (*n* = 32) in The Cancer Genome Atlas database. Kaplan–Meier analysis was used to verify the relationship between CYTL1 expression and overall survival (OS) and disease-specific survival (DSS) based on the clinical characteristics and subgroups of patients with STAD. Furthermore, univariate and multivariate Cox regression analyses were used to verify the outcome variables of OS and DSS in patients with STAD. Receiver operating characteristic curves were used to test the predictive power of CYTL1. The biological functions and signaling pathways of CYTL1 were determined using gene set enrichment analysis (GSEA), and the immune infiltration patterns of CYTL1 and correlation of immune-related markers were analyzed using single-sample GSEA (ssGSEA) and an estimate algorithm.

**Results:**

In our research, low CYTL1 expression (tumor vs. normal) was noted in patients with STAD. High CYTL1 expression was detrimental to OS and DSS and had good diagnostic performance (AUC = 0.731). In the subtype analysis of STAD, T3 and T4 stages, N0 and N1 stages, M0 stage, gender (female), and age (≤65 years) showed different performances between OS and DSS. Univariate and multivariate Cox analyses identified CYTL1 as an independent factor, and logistic regression analysis indicated that CYTL1 was associated with M stage (OR = 3.406) and sex (OR = 1.535). GSEA of the differential genes of CYTL1 showed the possible involvement of immunity. ssGSEA and estimation algorithms were used to further evaluate whether immune cells were closely related to CYTL1 expression, and many markers of immune cells also had statistical significance with the expression of CYTL1.

**Conclusion:**

CYTL1 may, thus, act as an independent prognostic factor for STAD and regulate STAD progression by affecting the immune microenvironment.

## 1. Introduction

Over 95% of gastric cancers are adenocarcinomas. Gastric cancer is the third leading cause of death worldwide, and over 95% of gastric cancers are stomach adenocarcinoma (STAD) [1, 2]. The survival rate of patients with advanced gastric cancer is very low, and the clinical symptoms of patients with early STAD are not obvious, resulting in delayed diagnosis [3, 4]. The treatment of STAD is usually based on TNM staging, and the treatment methods (surgery and chemotherapy) are determined accordingly. However, studies have confirmed that STAD shows strong heterogeneity in treatment, and developing a clinical treatment plan is an urgent problem that needs to be solved [5–8]. Therefore, finding biomarkers with high specificity and sensitivity is important for the early clinical diagnosis and prognostic evaluation of patients with STAD.

Cytokine-like protein 1 (CYTL1) [9], also known as C17, is a 136-amino acid protein secreted by CD34 hematopoietic cells. CYTL1 is closely related to inflammation [10, 11], is highly expressed in cartilage tissue [12], prevents inflammatory arthritis and joint destruction, and inhibits the expression of inflammation-related factors. Wen et al. [13] detected CYTL1 levels in 10 neuroblastoma cell lines and patient samples and showed that low CYTL1 expression inhibited the proliferation, migration, and invasion of human neuroblastoma SH-SY5Y cells. Wang et al. [14] concluded through bioinformatics that CYTL1 is usually hypermethylated in breast cancer and lung cancer; high CYTL1 expression has no significant effect on the proliferation of lung cancer but inhibits migration and invasion of lung cancer. CYTL1 expression was also confirmed to inhibit tumor metastasis in a breast cancer albino mouse strain BALB/cArc. Although Nie et al. [15] found that the 5-year overall survival rate of gastric cancer can be predicted by constructing a seven-gene prognostic signature, the specific functional mechanism and pathway are still unknown.

In this study, we identified the expression pattern of CYTL1 and assessed its prognostic ability in The Cancer Genome Atlas (TCGA) database. We uncovered the underlying mechanism, signaling pathway, and immune microenvironment of CYTL1 in STAD through multiple bioinformatics analyses and suggested that CYTL1 may act as an independent prognostic factor for STAD and regulate STAD progression by modulating the immune microenvironment.

## 2. Methods

### 2.1. TCGA Database Data Collection and Analysis

TCGA (https://portal.gdc.cancer.gov/) RNA-seq data and patient clinical data in HTSeq-FPKM format in STAD project [16], normal samples (*n* = 32), tumor samples (*n* = 375), and clinical data including age, sex, tumor lymph node (TNM) stage, survival status, and time were collected. In addition, STAD-paired samples (tumor tissues and paracancerous normal tissues in patients with STAD) were also retained (*n* = 27). The clinical data of patients with STAD are listed in Table S1.

### 2.2. Survival Analysis (Kaplan–Meier Analysis) in STAD

The value of CYTL1 for STAD survival was determined using Kaplan–Meier analysis. The “survminer” package (version 0.4.9) (for visualization) and the “survival” package (version 3.2-10) (for statistical analysis of survival data) in R were used for a series of subsequent analyses. Patients with STAD were grouped according to the median expression value of CYTL1, and survival rates (high and low groups) were compared, including subgroup analysis (T stage: T3 and T4; N stage: N0 and N1; M stage: M0; gender: female; age: ≤65 years), and the prognostic parameters analyzed included overall survival (OS) and disease-specific survival (DSS). The threshold was set at *p* < 0.05.

### 2.3. Univariate and Multivariate Cox Regression Analyses

The relationship between STAD clinical variables and prognosis was investigated using univariate and multivariate Cox regression analyses. The threshold for univariate analysis was set as 0.05, and the threshold for multivariate analysis was set as 0.01. The “coxph” function of the R “survival” package (version 3.2-10) was used for the Cox regression analyses. OS was selected as a prognostic factor. The clinical variables included in the analysis were T stage, N stage, M stage, age, sex, and CYTL1 expression levels.

### 2.4. Evaluation of Diagnostic Value: Receiver Operating Characteristic (ROC) Curve Analysis

ROC curve analysis of CYTL1 expression data was performed using the “pROC” package (version 1.17.0.1) and visualized using the “ggplot2” package (version 3.3.3). Predictive outcome parameters were defined as the clinical status (tumor vs. normal). In the ROC curve analysis, the *x*-axis represents the false-positive rate (FPR) and the *y*-axis represents the true-positive rate (TPR). The area under the curve (AUC) is the area enclosed by the ROC curve and coordinate axis. The closer the AUC is to 1, the better the diagnostic performance.

### 2.5. Gene Set Enrichment Analysis (GSEA) and Immune Enrichment Analysis

The “clusterProfiler” package (version 3.14.3) was used for exploration and GSEA in STAD. The species used was *Homo sapiens*. Differential gene expression analysis (DESeq2, version 1.26.0) was performed between datasets with low or high CYTL1 mRNA expression (log2foldchange ≥ 2, adjusted *p* value (*p*.adjust) < 0.05), and the differential genes were used for GSEA. Phenotypes were determined based on CYTL1 expression levels using TCGA database. An annotated gene set (c5.all.v7.2.symbols.gmt, c2.cp.v7.2.symbols.gmt) was used as the reference gene set. The normalized enrichment score (NES), nominal *p* value, and false discovery rate (FDR) *q*-value indicated the importance of the association between gene sets and pathways, and enrichment was considered significant if FDR < 0.25 and *p*.adjust < 0.05. Immune infiltration was analyzed as follows: normalized STAD gene expression data were compared to gene sets using the “GSVA” package (version 1.34.0). Single-sample GSEA (ssGSEA) was used to classify gene sets with common biological functions, chromosomal localization, and physiological regulation. These gene sets included 782 genes used to predict the abundance of 28 tumor-infiltrating immune cells (TIICs) in a single tissue sample (http://software.broadinstitute.org/gsea/msigdb/index.jsp). The identified immune cells included activated dendritic cells (aDCs), B cells, CD8 T cells, cytotoxic T cells, DCs, eosinophils, immature DCs (iDCs), macrophages, mast cells, neutrophils, NK CD56^bright^ cells, NK CD56^dim^ cells, NK cells, plasmacytoid DCs (pDCs), T cells, T helper cells, T central memory cells (Tcm), T effector memory cells (Tem), T follicular helper cells (Tfh), T gamma delta cells (Tgd), Th1 cells, Th17 cells, Th2 cells, and Treg cells. The STAD expression profile data from the CYTL1 high- and low-expression groups were compared with the gene sets to analyze the differences in immune cells between the two groups, and the Wilcoxon rank sum test and Spearman correlation were used; *p* < 0.01 was considered statistically significant. The “estimate” package (version 1.0.13), including StromalScore, ImmuneScore, and ESTIMATEScore, was used to analyze the immune infiltration patterns of the samples.

### 2.6. Statistical Analysis

Differences in CYTL1 expression between STAD tissues and normal tissues were compared using the Mann–Whitney *U* test, differences between STAD tissues and paracancerous tissues were identified by paired sample *t*-test, and analysis of CYTL1 between subgroups was performed using multiple hypothesis testing (Dunn's test). Logistic regression was used to analyze the inferential power of CYTL1 expression (low and high as dichotomous variables) for clinical variables. “ggplot2” (version 3.3.3) was used for visualization. The chi-squared (*χ*^2^) test was used to assess the correlation between CYTL1 expression levels and STAD clinicopathological parameters. A method provided by the “survminer” package utilizes the different expression values of molecules as the cut-off, and here, the cut-off value corresponding to the smallest *p* value was used as the cut-off for grouping (age = 65 years as a threshold). R version 4.0.3 (R Core Team 2020, https://www.R-project.org/) was used for data preprocessing, and Spearman's correlation was used to assess the correlation of certain variables, with *p* < 0.05 indicating statistical significance.

## 3. Result

### 3.1. Expression and Prognosis of CYTL1 in STAD

**Figure 1:**
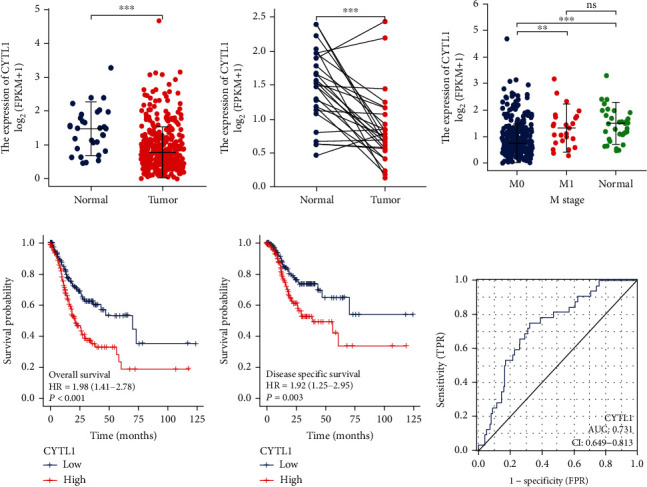
CYTL1 expression in STAD samples. (a) CYTL1 expression pattern in TCGA STAD samples (normal = 32, tumor = 375). (b) CYTL1 expression level in STAD paired samples (*n* = 27). (c) STAD (M stage) samples with different CYTL1 expression levels (M0 = 330 vs. M1 = 25 vs. normal = 32). (d) Kaplan–Meier curve analysis of CYTL1 expression patterns (high vs. low) associated with overall survival. (e) Kaplan–Meier curve analysis of disease-specific survival. (f) Receiver operating characteristic (ROC) curve of CYTL1 in patients with STAD. FPKM: fragments per kilobase per million; HR: hazard ratio. ns, *p* ≥ 0.05; ^∗^*p* < 0.05; ^∗∗^*p* < 0.01; ^∗∗∗^*p* < 0.001.

We found that CYTL1 was underexpressed in tumor tissues (*n* = 375) relative to that in normal tissues (*n* = 32) (*p* ≤ 0.001) (Figure 1(a)); to exclude experimental bias, we chose paired sample (*n* = 27) analysis. Similarly, the expression level of CYTL1 in tumor tissues was lower than that in normal tissues (*t* = −4.918, *p* ≤ 0.001) (Figure 1(b)). In addition, we found no significant difference in CYTL1 expression in T1–T4 and N0–N3 (*p* > 0.05) (Figure S1A-B), while CYTL1 expression in M0 stage patients was significantly lower than that in M1 stage patients (*p*.adjust = 0.006) and M0 vs. normal (*p*.adjust ≤ 0.001), but no significance in M1 vs. normal (*p*.adjust = 1.000) (Figure 1(c)). High CYTL1 expression promotes the progression of M0 patients to M1 patients. The corresponding survival curve results (Figure 1(d)) indicated that the survival time of the high CYTL1 expression group was significantly lower than that of the low CYTL1 expression group (HR, 1.97: 1.71–2.78, *p* ≤ 0.001), which was consistent with the results of the M stage. In addition, the high CYTL1 expression group (PFI) had a shorter survival time than the low CYTL1 expression group (HR, 1.47: 1.03–2.09, *p* = 0.035) (Figure 1(e)). ROC curve analysis of CYTL1 expression in STAD indicated that the predictive power of CYTL1 was accurate (AUC = 0.731, CI = 0.649–0.813). These results indicated that high CYTL1 expression resulted in a low survival rate of patients with STAD and the possible mechanism involved in metastasis.

### 3.2. OS and DSS between Clinical Characteristics of Patients with STAD and CYTL1

**Figure 2:**
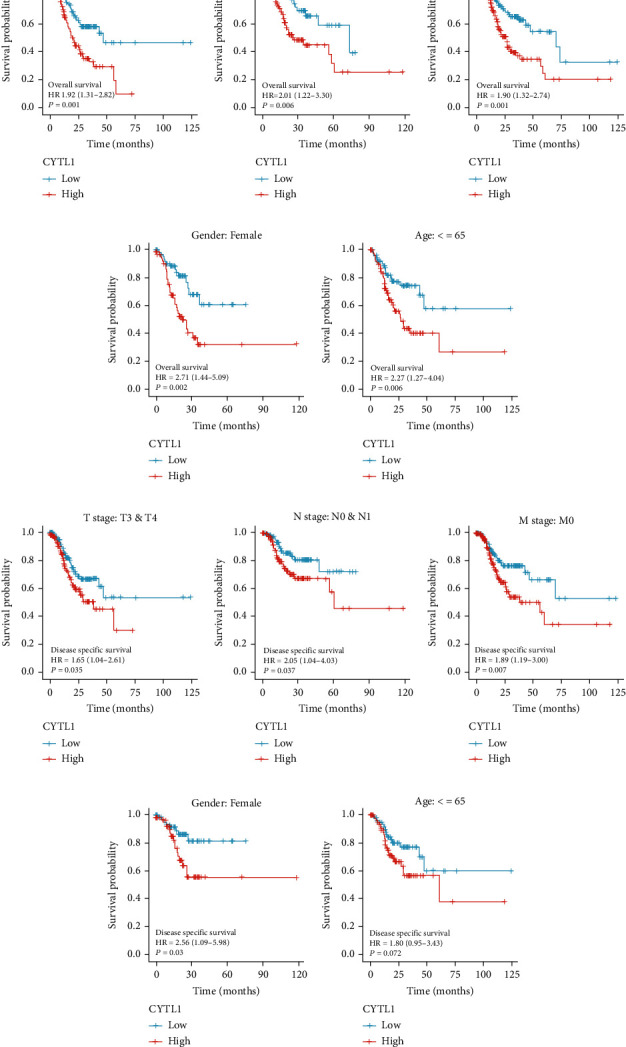
CYTL1 expression level in STAD clinical characteristics. (a–e) Kaplan–Meier analysis of CYTL1 expression levels associated with overall survival in STAD subtypes (T stage: T3 and T4; N stage: N0 and N1; M stage: M0; gender: female; age: ≤65 years). (f–j) Kaplan–Meier analysis of CYTL1 expression levels associated with disease-specific survival in STAD subtypes (T stage: T3 and T4; N stage: N0 and N1; M stage: M0; gender: female; age: ≤65 years). A *p* value < 0.05 was considered statistically significant.

To continue exploring the relationship between clinical features and CYTL1 expression, we analyzed the survival curves of different subgroups. The Kaplan–Meier (KM) curve results (Figures 2(a)–2(j)) indicated that the OS time of the high CYTL1 expression group was consistently lower than that of the low-expression group (T stage: T3 and T4; N stage: N0 and N1; M stage: M0; gender: female; age: ≤65 years; *p* < 0.05). Similarly, most of the high-expression groups had lower DSS than the low-expression groups (T stage: T3 and T4; N stage: N0 and N1; M stage: M0; gender: female; *p* < 0.05); age (≤65 years; *p* > 0.05) was not statistically significant. To further understand the relationship between CYTL1 and STAD clinical variables (Table S2), we summarized the correlation between CYTL1 expression level and STAD clinical variables. CYTL1 expression level was significantly correlated with M stage (*p* = 0.013). Logistic regression analysis showed that the reduction in CYTL1 expression level in STAD was associated with M stage (OR = 3.406 for M1 vs. M0) and sex (OR = 1.535 for male vs. female) (Table 1).

### 3.3. STAD Prognostic Model

To identify the prognostic factors for STAD, we used univariate and multivariate Cox regression models to screen for STAD OS and clinical variables related to DSS. Univariate cox and OS correlation analysis (Table 2) showed the following: T stage (*n* = 266, HR: 1.719, 1.131–2.612, *p* = 0.011), N stage (*n* = 148, HR: 1.650, 1.182–2.302, *p* = 0.003), M stage (*n* = 25, HR: 2.254, 1.295–3.924, *p* = 0.004), age (>65 years; *n* = 204, HR: 1.620, 1.154–2.276, *p* = 0.005), and CYTL1 expression (*n* = 185, HR: 1.981, 1.410–2.782, *p* ≤ 0.001). Multivariate cox results showed the following: N stage (HR: 1.628, 1.147–2.311, *p* = 0.006), M stage (HR: 2.233, 1.249–3.993, *p* = 0.007), age (HR: 1.856, 1.293–2.663, *p* ≤ 0.001), and CYTL1 expression (HR: 1.995, 1.393–2.857, *p* ≤ 0.001) (Table 3). The results of univariate and multivariate Cox analyses of DSS are as follows: T stage (*p* = 0.010), N stage (*p* ≤ 0.001), M stage (*p* = 0.012), and CYTL1 expression (*p* = 0.003) and N stage (*p* = 0.002) and CYTL1 expression (*p* = 0.005), respectively. These results indicated that CYTL1 is an important independent predictor of STAD.

### 3.4. Identification of CYTL1-Related Functions and Signaling Pathways

**Figure 3:**
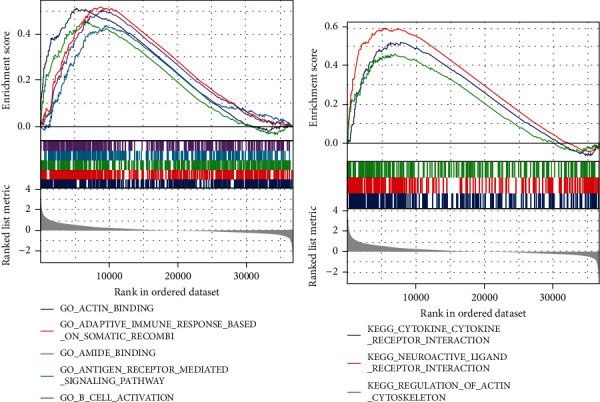
GSEA for CYTL1-mediated STAD. (a) GSEA (Gene Ontology) for differentially expressed genes related to CYTL1 expression level. Reference gene set: c5.all.v7.2.symbols.gmt in MSigDB. Species: *Homo sapiens*. (b) GSEA (Kyoto Encyclopedia of Genes and Genomes) for differentially expressed genes related to CYTL1 expression level. Reference gene set: c2.cp.v7.2.symbols.gmt in MSigDB. Species: *Homo sapiens*. False discovery rate (FDR) < 0.25 and *p*.adjust < 0.05 were the conditions for significant enrichment.

The above results preliminarily identified CYTL1 as an independent prognostic factor for STAD and may be used as a prognostic indicator for clinical diagnosis. Next, we explored the potential mechanisms and signaling pathways of CYTL1-mediated STAD using GSEA. All TCGA samples were grouped according to CYTL1 for high and low expression, and differentially expressed genes were identified by DESeq2 as a source for GSEA (threshold: log2foldchange ≥ 2, *p*.adjust < 0.05), and according to NES, FDR and *p*.adjust values identified significantly enriched signaling pathways. In this study (Figures 3(a) and 3(b)), functions were enriched in actin binding, adaptive immune response based on somatic recombination of immune receptors built from immunoglobulin superfamily domains, amide binding, and antigen receptor-mediated signaling pathways. The pathways were enriched in cytokine–cytokine receptor interaction, neuroactive ligand–receptor interaction, and actin cytoskeleton regulation.

### 3.5. Correlation between Immune Cell Infiltration and CYTL1

**Figure 4:**
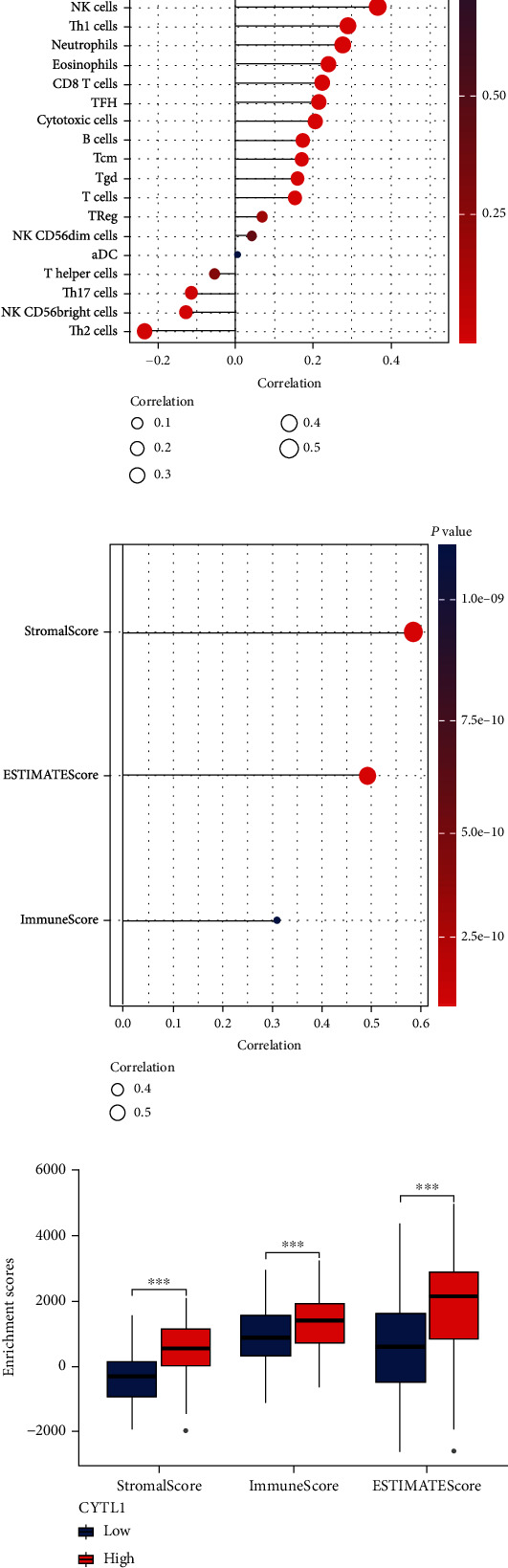
Immune cells and immune infiltration by ssGSEA and estimate analysis were correlated to CYTL1 expression. (a) Twenty-four immune cells (activated dendritic cells (aDCs), B cells, CD8 T cells, cytotoxic T cells, DCs, eosinophils, immature DCs (iDCs), macrophages, mast cells, neutrophils, NK CD56^bright^ cells, NK CD56^dim^ cells, NK cells, plasmacytoid DCs (pDCs), T cells, T helper cells, T central memory cells (Tcm), T effector memory cells (Tem), T follicular helper cells (Tfh), T gamma delta cells (Tgd), Th1 cells, Th17 cells, Th2 cells, and Treg cells) were correlated with CYTL1 expression. Red represents small *p* value, blue represents high *p* value, and large circle indicates high correlation. (b) Spearman analysis of CYTL1 expression and ImmuneScore, StromalScore, and ImmuneScore. Red represents small *p* value, blue represents high *p* value, and large circle indicates high correlation. (c) Enrichment scores of CYTL1 (high- and low-expression groups) in ImmuneScore, StromalScore, and ESTIMATEScore. ns, *p* ≥ 0.05; ^∗^*p* < 0.05; ^∗∗^*p* < 0.01; ^∗∗∗^*p* ≤ 0.001.

Based on the above conclusions, CYTL1 was closely related to the M stage, and from the GSEA results, the regulatory mechanism of CYTL1 was mediated by immunity and receptors. Previous studies [17, 18] have confirmed that immune regulation is crucial for the development of cancer. We continued to calculate the enriched infiltration of 24 immune cells [19] with CYTL1 by ssGSEA and additionally evaluated the immune infiltration score, matrix score, and estimate score of STAD samples by the estimate algorithm [20]. ssGSEA results (Figure 4(a) and Table S3) suggested that CYTL1 was associated with B cells (*r* = 0.172, *p* ≤ 0.001), CD8 T cells (*r* = 0.222, *p* ≤ 0.001), cytotoxic cells (*r* = 0.205, *p* ≤ 0.001), DCs (*r* = 0.423, *p* ≤ 0.001), eosinophils (*r* = 0.238, *p* ≤ 0.001), iDCs (*r* = 0.374, *p* ≤ 0.001), macrophages (*r* = 0.441, *p* ≤ 0.001), mast cells (*r* = 0.468, *p* ≤ 0.001), neutrophils (*r* = 0.275, *p* ≤ 0.001), NK CD56^bright^ cells (*r* = 0.128, *p* = 0.013), NK cells (*r* = 0.364, *p* ≤ 0.001), pDCs (*r* = 0.511, *p* ≤ 0.001), T cells (*r* = 0.152, *p* = 0.003), Tcm (*r* = 0.169, *p* ≤ 0.001), Tem (*r* = 0.376, *p* ≤ 0.001), TFH (*r* = 0.214, *p* ≤ 0.001), Tgd (*r* = 0.158, *p* = 0.002), Th1 cells (*r* = 0.287, *p* ≤ 0.001), Th17 cells (*r* = −0.115, *p* = 0.026), and Th2 (*r* = −0.234, *p* ≤ 0.001), and Table 4 showed that CYTL1 (high and low group) expression pattern was significantly associated with most immune cells. In addition, the estimate algorithm processed the results (Figures 4(b) and 4(c)) of ImmuneScore (*r* = 0.309, *p* ≤ 0.001), StromalScore (*r* = 0.584, *p* ≤ 0.001), ImmuneScore (low vs. high, 95% CI: 261.040–626.344, *p* ≤ 0.001), and StromalScore (low vs. high, 95% CI: 736.268–1048.961, *p* ≤ 0.001) and continued to explore the correlation of immune cell markers and CYTL1 expression. Interestingly, most of the STAD immune biomarkers were positively correlated with the expression of CYTL1, B cells (MS4A1, CD79A, and TCL1A), and cytotoxic cells (GZMK, KLRF1); DCs (HLA-DRA, HLA-DPB1, and HLA-DPA1), eosinophils (CCR3, IL5RA, and SYNJ1), iDCs (CD1E, CD1A), macrophages (CCL7, FN1, and PPBPCD163), mast cells (CMA1, TPSAB1, and MS4A2), neutrophils (FPR2, CXCR1), pDCs (IL3RA), T cells (CD2, CD96, and TRBC1), Tcm (USP9Y, FOXP1, CCR2, and FLI1), TFH (CHI3L2, CHGB), Th1 cells (STAT4, CD38, and LTA), and Th2 (PTGIS, MB) were positively correlated and CD8 T cells (PRR5, SF1); NK cells (CDC5L) were negatively correlated. The above conclusions suggest that CYTL1 regulates STAD through immune infiltration.

## 4. Discussion

Our study confirmed the correlation between CYTL1 and the M stage of STAD, and this mechanism may be caused by the immune environment or immune infiltration. As a secreted protein produced by hematopoietic cells, CYTL1 has been confirmed in various studies. CYTL1, which is produced by the bone marrow and umbilical cord blood, may have immune functions. In previous studies [21, 22], CYTL1 was shown to be involved in cartilage formation and inhibited inflammation. In addition, Xue et al. [9] stated that CYTL1 acts as a tumor suppressor and maintains intracellular metabolic homeostasis in tumors, although Wang et al. [14] initially explored the expression patterns of CYTL1 in breast, prostate, lung, and gastric cancers through bioinformatics. However, no further research has been conducted to confirm the mechanism of CYTL1 in STAD. Our study is the first to confirm that CYTL1 is involved in immune regulation and promotes STAD progression.

We applied bioinformatics methods to identify the expression pattern of STAD patients; although CYTL1 was low expressed in STAD (relative to normal samples), high expression of CYTL1 often predicted poor prognosis (including OS and DSS). It was interesting that the expression level of CYTL1 was in M1 stage STAD patients, and CYTL1 was also highly expressed in normal tissues, while the expression level of M0 stage was relatively low, which also indicated that in the process of STAD patient metastasis, the expression of CYTL1 will increase and accelerate tumor progression (in our experience, M1 patients also meant extremely poor survival); we speculated that high expression of CYTL1 in STAD patients before tumor metastasis was beneficial to patient survival, and during tumor metastasis, CYTL1 somehow promoted the progression of STAD.

Wang et al. [23] confirmed that CYTL1 exhibits chemotactic activity. By combining with CCR2B, it strengthened the attraction of monocytes and macrophages and expanded its immunobiological function. In a research by Wang et al. [14], it acted as a cytokine that inhibited tumor metastasis and tumor cell progression. In our study, CYTL1 was confirmed as an independent prognostic factor of STAD by univariate and multivariate Cox regression, and the potential biological functions and signaling pathways of CYTL1 were analyzed using GSEA. As shown in many previous studies, CYTL1 is involved in immune regulation, and immune regulation is involved in STAD M-stage progression, for which we continued to explore the context of CYTL1-mediated immune infiltration.

The tumor microenvironment is complex and mainly composed of tumor cells, tumor-infiltrating cells, and extracellular mediators. The treatment of tumors can differ due to heterogeneity and changes in the tumor microenvironment. Hong et al. [24] showed that Tgd cells, mast cells, Th2 cells, and Th1 cells were related to the prognosis of STAD; Wang et al. [25] reported that immune cell infiltration (Th2 and mast cells) was closely related to the survival of STAD patients, and Ma et al. [26] reported that metastasis of STAD was altered by enhancing epithelial-mesenchymal changes. In our study, previous GSEA results also mentioned the relationship between CYTL1 and immunity. We speculated that CYTL1 accelerated progression from M0 to M1 stage by changing the immune environment. Immune infiltration in STAD patients was measured, and as expected, CYTL1 was closely associated with B cells, CD8 T cells, cytotoxic cells, dendritic cells, etc. (*r* = 0.309, *p* ≤ 0.001). High expression of CYTL1 indicated a high degree of immune infiltration. We further analyzed the correlation between immune cell markers and CYTL1, which was consistent with our results. This evidence suggests that CYTL1 promotes the metastasis and progression of STAD by activating immune activity.

In our study, we confirmed that high expression of CYTL1 in STAD patients with M1 metastasis activates immune regulation and shortens the survival time of STAD patients. However, in nonmetastatic STAD patients, the expression of CYTL1 was suppressed, which cannot activate immunity. Our future research will be devoted to mechanistic studies of CYTL1 in metastatic and nonmetastatic STAD patients, and we hope to corroborate our findings from in vivo and in vitro experiments. In conclusion, the expression of CYTL1 is closely related to the prognosis of STAD, and CYTL1 may affect the survival and prognosis of STAD by regulating immune infiltration. Our study provides a new understanding of immunotherapy for patients with STAD.

## 5. Conclusion

Our study suggests that CYTL1 may act as an independent prognostic factor for STAD and may regulate STAD progression by modulating the immune microenvironment.

## Figures and Tables

**Table 1:** CYTL1 expression correlated with clinical characteristics (logistic regression analysis).

Characteristics	Total (*N*)	Odds ratio (OR)	*p* value
T stage (T3 & T4 vs. T1 & T2)	367	1.347 (0.849-2.147)	0.208
N stage (N1 & N2 & N3 vs. N0)	357	1.215 (0.776-1.909)	0.395
M stage (M1 vs. M0)	355	3.406 (1.400-9.547)	*0.011*
Gender (male vs. female)	375	1.535 (1.004-2.354)	*0.048*
Age (>65 vs. ≤65)	371	0.827 (0.548-1.246)	0.365

CYTL1: cytokine-like protein 1; italic indicates *p* < 0.05.

**Table 2:** Univariate and multivariate analysis of CYTL1 and other clinical characteristics for OS.

Characteristics	Total (*N*)	Univariate analysis		Multivariate analysis	
		Hazard ratio (95% CI)	*p* value	Hazard ratio (95% CI)	*p* value
T stage	362				
T1 & T2	96	Reference			
T3 & T4	266	1.719 (1.131-2.612)	**0.011**	1.426 (0.904-2.249)	0.127
N stage	352				
N0 & N1	204	Reference			
N3 & N2	148	1.650 (1.182-2.302)	**0.003**	1.628 (1.147-2.311)	*0.006*
M stage	352				
M0	327	Reference			
M1	25	2.254 (1.295-3.924)	**0.004**	2.233 (1.249-3.993)	*0.007*
Gender	370				
Female	133	Reference			
Male	237	1.267 (0.891-1.804)	0.188		
Age	367				
≤65	163	Reference			
>65	204	1.620 (1.154-2.276)	**0.005**	1.856 (1.293-2.663)	*<0.001*
CYTL1	370				
Low	185	Reference			
High	185	1.981 (1.410-2.782)	**<0.001**	1.995 (1.393-2.857)	*<0.001*

Cl: confidence interval; CYTL1: cytokine-like protein 1; OS: overall survival; bold means *p* < 0.05; italic means *p* < 0.01.

**Table 3:** Univariate and multivariate analysis of CYTL1 and other clinical characteristics for DSS.

Characteristics	Total (*N*)	Univariate analysis		Multivariate analysis	
		Hazard ratio (95% CI)	*p* value	Hazard ratio (95% CI)	*p* value
T stage	345				
T1 & T2	90	Reference			
T3 & T4	255	2.089 (1.192-3.660)	**0.010**	1.701 (0.935-3.096)	0.082
N stage	334				
N0 & N1	192	Reference			
N3 & N2	142	2.110 (1.378-3.231)	**≤0.001**	1.983 (1.276-3.082)	*0.002*
M stage	333				
M0	311	Reference			
M1	22	2.438 (1.221-4.870)	**0.012**	1.952 (0.963-3.957)	0.064
Gender	349				
Female	125	Reference			
Male	224	1.573 (0.985-2.514)	0.058	1.638 (1.007-2.667)	0.047
Age	346				
≤65	160	Reference			
>65	186	1.211 (0.797-1.840)	0.371		
CYTL1	349				
Low	177	Reference			
High	172	1.922 (1.252-2.951)	**0.003**	1.905 (1.218-2.978)	*0.005*

Cl: confidence interval; CYTL1: cytokine-like protein 1; DSS: disease-specific survival; bold means *p* < 0.05; italic means *p* < 0.01.

**Table 4:** Twenty-four immune cells and CYTL1 expression analysis.

Group	CYTL1 (low, *n*)	CYTL1 (high, *n*)	*t*	95% CI	*p* value
aDC	Low [187]	High [188]	-0.365	-0.028-0.019	0.716
B cells	Low [187]	High [188]	2.965	0.011-0.053	*0.003*
CD8 T cells	Low [187]	High [188]	3.115	0.003-0.012	*0.002*
Cytotoxic cells	Low [187]	High [188]	2.765	0.008-0.048	*0.006*
DC	Low [187]	High [188]	6.461	0.048-0.090	*≤0.001*
Eosinophils	Low [187]	High [188]	4.179	0.009-0.024	*≤0.001*
iDC	Low [187]	High [188]	6.338	0.025-0.048	*≤0.001*
Macrophages	Low [187]	High [188]	7.500	0.037-0.064	*≤0.001*
Mast cells	Low [187]	High [188]	8.266	0.056-0.091	*≤0.001*
Neutrophils	Low [187]	High [188]	4.756	0.025-0.061	*≤0.001*
NK CD56bright cells	Low [187]	High [188]	-0.963	-0.017-0.006	0.336
NK CD56dim cells	Low [187]	High [188]	-0.488	-0.019-0.011	0.626
NK cells	Low [187]	High [188]	6.657	0.018-0.032	*≤0.001*
pDC	Low [187]	High [188]	8.939	0.069-0.108	*≤0.001*
T cells	Low [187]	High [188]	2.209	0.003-0.049	0.028
T helper cells	Low [187]	High [188]	-1.149	-0.009-0.002	0.251
Tcm	Low [187]	High [188]	2.503	0.002-0.018	0.013
Tem	Low [187]	High [188]	6.173	0.017-0.033	*≤0.001*
TFH	Low [187]	High [188]	3.617	0.008-0.026	*≤0.001*
Tgd	Low [187]	High [188]	2.079	0.001-0.020	0.038
Th1 cells	Low [187]	High [188]	4.375	0.014-0.036	*≤0.001*
Th17 cells	Low [187]	High [188]	-1.427	-0.041-0.007	0.154
Th2 cells	Low [187]	High [188]	-4.112	-0.021-0.008	*≤0.001*
Treg	Low [187]	High [188]	1.192	-0.011-0.043	0.234

Activated dendritic cells (aDCs), B cells, CD8 T cells, cytotoxic T cells, DCs, eosinophils, immature DCs (iDCs), macrophages, mast cells, neutrophils, NK CD56^bright^ cells, NK CD56^dim^ cells, NK cells, plasmacytoid DCs (pDCs), T cells, T helper cells, T central memory cells (Tcm), T effector memory cells (Tem), T follicular helper cells (Tfh), T gamma delta cells (Tgd), Th1 cells, Th17 cells, Th2 cells, and Treg cells. 95% CI: confidence interval. A *p* value < 0.01 was considered statistically significant (italic).

## Data Availability

The data of this study were downloaded and compiled from the GEO database (https://www.ncbi.nlm.nih.gov/gds/?term=); data used to support the results of this study were obtained from the corresponding authors.

## Supplementary Materials

Supplementary 1 Figure S1: A CYTL1 expression in T stage (T1 and T2 and T3 and T4). B CYTL1 expression in N stage (N0 and N1 and N2 and N3). ns, *p* ≥ 0.05; ^∗^*p* < 0.05; ^∗∗^*p* < 0.01; ^∗∗∗^*p* ≤ 0.001.

Supplementary 2 Table S1: clinical characteristics of STAD patients (*n* = 375).

Supplementary 3 Table S2: relationship between expression of CYTL1 and various clinical characteristics.

Supplementary 4 Table S3: correlation of CYTL1 expression level with 24 immune cells.
