# Supplementary figures and images for: Cytokine-Like Protein 1 (CYTL1) as a Key Target of M-Stage Immune Infiltration in Stomach Adenocarcinoma
Source: Biomed Res Int. 2023 Feb 13;2023:2926218. doi: 10.1155/2023/2926218 (PMC9941682; doi:10.1155/2023/2926218)

A

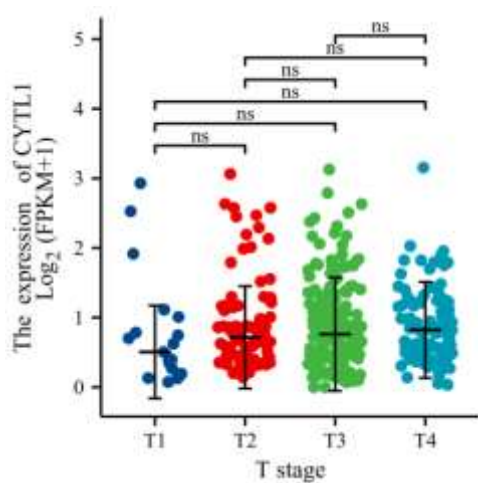

B

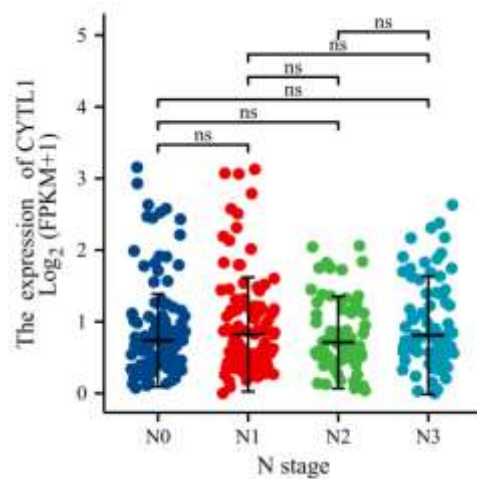

**Figure S1:**A.CYTL1 expression in T stage(T1&T2&T3&T4).**B.** CYTL1 expression in N stage(N0&N1&N2&N3). ns,  $p \geq 0.05$ ; \*,  $p < 0.05$ ; \*\*,  $p < 0.01$ ; \*\*\*,  $p \leq 0.001$ .

Supplement: Supplementary 1 — Figure S1: A CYTL1 expression in T stage (T1 and T2 and T3 and T4). B CYTL1 expression in N stage (N0 and N1 and N2 and N3). ns, p ≥ 0.05; ∗p < 0.05; ∗∗p < 0.01; ∗∗∗p ≤ 0.001. [file 2926218.f1.pdf]
