# Supplementary material for: Cytokine-Like Protein 1 (CYTL1) as a Key Target of M-Stage Immune Infiltration in Stomach Adenocarcinoma
Source: Biomed Res Int. 2023 Feb 13;2023:2926218. doi: 10.1155/2023/2926218 (PMC9941682; doi:10.1155/2023/2926218)
Supplement: Supplementary 2 — Table S1: clinical characteristics of STAD patients (n = 375). [file 2926218.f2.docx]

**Table S1:**Clinical Characteristics of STAD patients(n=375)

| Characteristic | levels | Overall |
| --- | --- | --- |
| n |  | 375 |
| T stage, n (%) | T1 | 19 (5.2%) |
|  | T2 | 80 (21.8%) |
|  | T3 | 168 (45.8%) |
|  | T4 | 100 (27.2%) |
| N stage, n (%) | N0 | 111 (31.1%) |
|  | N1 | 97 (27.2%) |
|  | N2 | 75 (21%) |
|  | N3 | 74 (20.7%) |
| M stage, n (%) | M0 | 330 (93%) |
|  | M1 | 25 (7%) |
| Gender, n (%) | Female | 134 (35.7%) |
|  | Male | 241 (64.3%) |
| Age, median (IQR) |  | 67 (58, 73) |

IQR, interquartile range,STAD, Stomach adenocarcinoma;
