# Supplementary material for: Cytokine-Like Protein 1 (CYTL1) as a Key Target of M-Stage Immune Infiltration in Stomach Adenocarcinoma
Source: Biomed Res Int. 2023 Feb 13;2023:2926218. doi: 10.1155/2023/2926218 (PMC9941682; doi:10.1155/2023/2926218)
Supplement: Supplementary 3 — Table S2: relationship between expression of CYTL1 and various clinical characteristics. [file 2926218.f3.docx]

| Characteristic | Low expression of CYTL1 | High expression of CYTL1 | p |
| --- | --- | --- | --- |
| n | 187 | 188 |  |
| T stage, n (%) |  |  | 0.237 |
| T1 | 13 (3.5%) | 6 (1.6%) |  |
| T2 | 42 (11.4%) | 38 (10.4%) |  |
| T3 | 85 (23.2%) | 83 (22.6%) |  |
| T4 | 44 (12%) | 56 (15.3%) |  |
| N stage, n (%) |  |  | 0.600 |
| N0 | 60 (16.8%) | 51 (14.3%) |  |
| N1 | 48 (13.4%) | 49 (13.7%) |  |
| N2 | 40 (11.2%) | 35 (9.8%) |  |
| N3 | 33 (9.2%) | 41 (11.5%) |  |
| M stage, n (%) |  |  | **0.013** |
| M0 | 171 (48.2%) | 159 (44.8%) |  |
| M1 | 6 (1.7%) | 19 (5.4%) |  |
| Gender, n (%) |  |  | 0.061 |
| Female | 76 (20.3%) | 58 (15.5%) |  |
| Male | 111 (29.6%) | 130 (34.7%) |  |
| Age, mean ± SD | 66.24 ± 10.87 | 65.43 ± 10.44 | 0.464 |

**Table S2**:Relationship between expression of CYTL1 and viarous clinical characteristics.

SD, Standard Deviation;CYTL1, Cytokine-like protein 1.bold red means p<0.05.
