# Supplementary material for: Cytokine-Like Protein 1 (CYTL1) as a Key Target of M-Stage Immune Infiltration in Stomach Adenocarcinoma
Source: Biomed Res Int. 2023 Feb 13;2023:2926218. doi: 10.1155/2023/2926218 (PMC9941682; doi:10.1155/2023/2926218)
Supplement: Supplementary 4 — Table S3: correlation of CYTL1 expression level with 24 immune cells. [file 2926218.f4.docx]

| Immune Cells | Spearman | P vaule |
| --- | --- | --- |
| aDC | 0.004 | 0.934 |
| B cells | 0.172 | <0.001 |
| CD8 T cells | 0.222 | <0.001 |
| Cytotoxic cells | 0.205 | <0.001 |
| DC | 0.423 | <0.001 |
| Eosinophils | 0.238 | <0.001 |
| iDC | 0.374 | <0.001 |
| Macrophages | 0.441 | <0.001 |
| Mast cells | 0.468 | <0.001 |
| Neutrophils | 0.275 | <0.001 |
| NK CD56bright cells | -0.128 | 0.013 |
| NK CD56dim cells | 0.041 | 0.423 |
| NK cells | 0.364 | <0.001 |
| pDC | 0.511 | <0.001 |
| T cells | 0.152 | 0.003 |
| T helper cells | -0.054 | 0.294 |
| Tcm | 0.169 | <0.001 |
| Tem | 0.376 | <0.001 |
| TFH | 0.214 | <0.001 |
| Tgd | 0.158 | 0.002 |
| Th1 cells | 0.287 | <0.001 |
| Th17 cells | -0.115 | 0.026 |
| Th2 cells | -0.234 | <0.001 |
| TReg | 0.067 | 0.193 |

**Table S3,** Correlation of CYTL1 expression level with 24 immune cells

aDC(activated Dendritic Cells);CD8 T cells; Cytotoxic cells; DC; Eosinophils; iDC (immature DC); Macrophages; Mast cells; Neutrophils; NK CD56bright cells; NK CD56dim cells; NK cells; pDC(Plasmacytoid DC);Tcm(T central memory); Tem(T effector memory); Tfh(T follicular helper); Tgd(T gamma delta).red markers mean p<0.01
